# Linking Gene Fusions to Bone Marrow Failure and Malignant Transformation in Dyskeratosis Congenita

**DOI:** 10.3390/ijms25031606

**Published:** 2024-01-28

**Authors:** Ömer Güllülü, Benjamin E. Mayer, Fran Bačić Toplek

**Affiliations:** 1 Department of Structural Biology, St. Jude Children’s Research Hospital, Memphis, TN 38105, USA; 2 Computational Biology & Simulation, Technische Universität Darmstadt, 64287 Darmstadt, Germany; 3 Dipartimento di Bioscienze, Università degli Studi di Milano, 20133 Milano, Italy

**Keywords:** Dyskeratosis congenita, telomere disorders, bone marrow failure, genomic instability, gene fusions, RNA-Seq, polyampholytes

## Abstract

Dyskeratosis Congenita (DC) is a multisystem disorder intrinsically associated with telomere dysfunction, leading to bone marrow failure (BMF). Although the pathology of DC is largely driven by mutations in telomere-associated genes, the implications of gene fusions, which emerge due to telomere-induced genomic instability, remain unexplored. We meticulously analyzed gene fusions in RNA-Seq data from DC patients to provide deeper insights into DC’s progression. The most significant DC-specific gene fusions were subsequently put through in silico assessments to ascertain biophysical and structural attributes, including charge patterning, inherent disorder, and propensity for self-association. Selected candidates were then analyzed using deep learning-powered structural predictions and molecular dynamics simulations to gauge their potential for forming higher-order oligomers. Our exploration revealed that genes participating in fusion events play crucial roles in upholding genomic stability, facilitating hematopoiesis, and suppressing tumors. Notably, our analysis spotlighted a particularly disordered polyampholyte fusion protein that exhibits robust higher-order oligomerization dynamics. To conclude, this research underscores the potential significance of several high-confidence gene fusions in the progression of BMF in DC, particularly through the dysregulation of genomic stability, hematopoiesis, and tumor suppression. Additionally, we propose that these fusion proteins might hold a detrimental role, specifically in inducing proteotoxicity-driven hematopoietic disruptions.

## 1. Introduction

Dyskeratosis Congenita (DC) is a genetically heterogeneous, multisystem disorder that presents a spectrum of clinical manifestations, with bone marrow failure (BMF) being one of its most severe outcomes. Classically recognized by a tetrad of oral leukoplakia, nail dystrophy, reticular skin pigmentation, and pulmonary fibrosis, DC has become emblematic in the field of telomere biology [1].

The genetic underpinning of DC lies in its relationship with telomeres, the repetitive nucleotide sequences and protein complexes that cap and protect the ends of eukaryotic chromosomes. These telomeric regions safeguard the genome by preventing chromosomal end-to-end fusions, exonucleolytic degradation, and aberrant recombination events. Mutations in genes responsible for the synthesis, maintenance, and protection of telomeres disrupt the usual equilibrium of telomere length homeostasis. Defects in genes encoding for components of the telomerase complex, like TERT and TERC, or those encoding shelterin proteins, like TINF2, or even Dyskerin, encoded by the *DKC1* gene, have all been implicated in DC. The resultant telomere dysfunction is central to the pathogenesis of DC, culminating in the clinical phenotype of premature aging and organ dysfunction, with the bone marrow often being the most critically affected organ [1,2]. Cytogenetic studies have also revealed that both structural and numerical chromosomal aberrations are common manifestations in DC, playing a critical role in its pathology and influencing disease progression. These aberrations are also suggested to promote genomic rearrangements [3,4,5,6].

While strides have been made in identifying the genetic culprits of DC and understanding their role in telomere biology, the downstream genetic repercussions, like the occurrence of gene fusions due to genomic instability, remain relatively uncharted. In the context of DC and its resultant bone marrow failure, could gene fusions arising from telomere-induced genomic instability play a role in the pathogenesis? Recognizing and mapping these gene fusions could provide profound insights into disease progression and even potential therapeutic targets.

Here, we postulated that telomere dysfunction may induce genomic instability, leading to chromosomal rearrangements, specifically gene fusions. These fusions, commonly identified in diverse malignancies [7], might exacerbate the pathogenesis of BMF in DC. To address this hypothesis, we adopted a multifaceted approach to explore gene fusions in DC. By leveraging the power of next-generation sequencing and bioinformatic analyses, we aimed to elucidate the landscape of gene fusions in DC and highlight the importance of several high-confidence gene fusions potentially involved in BMF progression in DC. Concurrently, this endeavor extends to modeling the conformational and self-association dynamics of emergent highly disordered polyampholyte fusion proteins, probing their potential role in proteotoxicity-mediated hematopoietic anomalies.

## 2. Results

### 2.1. Identification of Gene Fusions Associated with Dyskeratosis Congenita

In this work, an extensive analysis of gene fusions was undertaken to discern their potential influence on Dyskeratosis Congenita (DC) pathogenesis. Utilizing next-generation RNA sequencing data from 219 lung epithelial cell samples—encompassing both DC patients and healthy individuals—280 novel, high-confidence gene fusions were identified that might be implicated in the DC phenotype (Suppl. Table S1). Comprehensive listings of gene fusion findings from DC patients (Suppl. Table S2) and healthy participants (Suppl. Table S3) are provided in the supplementary tables. Subsequent refinement, considering non-zero sequence coverages, split reads, and discordant mates, spotlighted 32 paramount in-frame gene fusions, as detailed in Table 1.

### 2.2. Genes Engaged in Fusion Events Predominantly Govern Genomic Integrity, Hematopoiesis, and Tumor Suppression

Interestingly, a significant portion of the genes implicated in fusion events (Table 1) play roles in preserving genomic stability. These include *CAMTA1* [9], *ESCO1* [10,11], *FOXO3* [12,13,14], *GOLGB1* [15], *MAP7* [16], *REV3L* [17], *RRM2B* [18], *USP38* [19], *VIM* [20], and *ZBTB38* [21]. They are involved in DNA replication, ensuring accurate genetic material duplication, and DNA repair mechanisms that fix damage. These genes also maintain chromosomal integrity and participate in wider genome maintenance tasks, including chromatin organization and cell cycle regulation. A disruption in these genes’ function, due to fusion events, can lead to genomic instability, a precursor to conditions like cancer.

Besides genomic stability, genes crucial for the homeostasis and function of platelets, hematopoietic cells, and leukocytes were also identified. Notable among these are *ARHGAP17* [22], *LEF1* [23], *LRRFIP1* [24,25], *METAP1* [26], *PNRC1* [27], *PTMA* [28], and *THBS1* [29]. These cells, each with a distinctive role, collectively maintain the body’s hemostatic homeostasis. When gene functions integral to the development, proliferation, or differentiation of these cells are disrupted, particularly due to fusion events, the repercussions can be profound. Such disruptions in key genes can skew the balance of hematopoiesis, derailing the normal production and function of these cells. For instance, LRRFIP1 serves as a key component of the platelet cytoskeleton, interacting with actin-remodeling proteins such as Flightless-1 and Drebrin [25]. Moreover, THBS1 influences platelet activation by modulating inhibitory cyclic adenosine monophosphate signaling [29]. The disruption of LRRFIP1 and THBS1 protein expression through gene fusions in DC could lead to adverse effects on platelet hemostasis, manifesting as dysregulated cytoskeletal architecture and impaired activation of platelets. Over time, this can culminate in bone marrow failure, wherein the marrow becomes unable to produce adequate numbers of vital blood cells, leading to a myriad of health complications, ranging from increased susceptibility to leukemia/ myelodysplastic syndromes (MDS), immunodeficiency, infections, and anemia.

Tumor suppressor genes are pivotal in regulating cell growth and preventing cancer. However, when involved in gene fusions, they can produce aberrant chimeric proteins that may lose their tumor-suppressing abilities, gain harmful functions, or exert dominant-negative effects. Such disruptions can lead to uncontrolled cell growth and contribute to cancer development [30]. Within the gene fusions identified in this research, many genes have been linked to tumor-suppressing activities. These include *ARHGAP17* [31], *CAMTA1* [32,33], *DAPK3* [34], *FOXO3* [35], *HSF2* [36], *LEF1* [37], *PALMD* [38], *PNRC1* [39], *QKI* [40], *RB1CC1* [41], *RBMS3* [42], *RGS2* [43], and *ZBTB38* [44]. In a twist of irony, while tumor suppressor genes are designed to combat cancer, their participation in gene fusions can inadvertently promote diseases like HNSCC (head and neck squamous cell carcinoma), cSCC (cutaneous squamous cell carcinoma), and AML (acute myeloid leukemia), as frequently observed in DC cases [45]. Supporting this, our overrepresentation network analysis of genes from gene fusion hits of DC samples (Suppl. Table S1) revealed a significant enrichment in specific TCGA cancer subtypes, including HNSCC and cSCC (Suppl. Figure S1).

**Figure 1:**
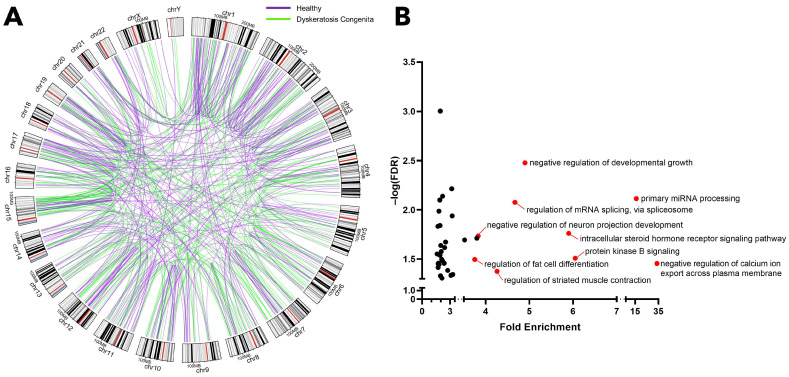
Heterogenous nature of gene fusions. (**A**) A comparative Circos plot illustrating the gene fusions identified in both DC-specific and healthy samples. (**B**) A Gene Ontology analysis of the gene fusions found in healthy samples. FDR: False Discovery Rate.

Furthermore, we investigated whether gene fusions specific to DC occur more frequently near the chromosomal ends, proximal to telomeric regions, given the known dysregulation of telomere end processing in DC patients. To explore this, we applied the same evaluation strategy used in Supplementary Table S1 to the gene fusion data from healthy samples. This analysis resulted in the creation of a high-confidence list of gene fusion events, as presented in Supplementary Table S4. This fusion data was further plotted in a Circos representation together with DC-specific data (Suppl. Table S1) for comparative visualization of the genomic locations and frequency of gene fusions between these two sets. The findings suggest that genomic rearrangements of both groups are highly heterogeneous and do not show a significantly enriched frequency of gene fusions located at telomeric regions in DC samples (Figure 1A).

Subsequently, a Gene Ontology analysis was conducted on the genes implicated in fusion events within healthy samples. This analysis aimed to ascertain whether the genes involved in DC-specific gene fusions are exclusively associated with roles in genomic stability, hematopoiesis, and tumor suppression. The results, illustrated in Figure 1B, revealed a significant enrichment of genes associated with processes like calcium ion export, miRNA processing, the steroid hormone pathway, developmental growth, mRNA splicing, neuron projection development, striated muscle contraction, and fat cell differentiation. Intriguingly, the identified pathways are not directly related to genomic stability, hematopoiesis, or tumor suppression, but are instead predominantly tissue-specific, involving the brain, muscle, endocrine, and adipose tissues. This suggests that the gene fusion events observed in healthy samples are likely non-specific to disease and lung tissue and may be non-expressive or non-functional in this context due to their tissue-specific nature.

**Figure 2:**
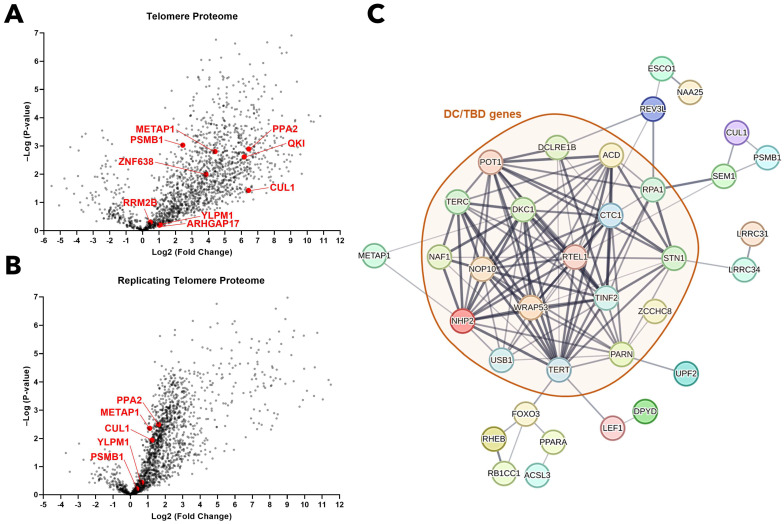
Genes forming fusions in DC are enriched in telomeric proteome and associates with known DC/TBD genes. (**A**) A volcano plot representation of the QTIP telomere proteome data from Lin et al. (2021) [46], showcasing the enriched fusion genes identified in Table 1. (**B**) A volcano plot representation of the QTIP-iPOND replicating telomere proteome data from Lin et al. (2021) [46], also highlighting the enriched fusion genes from Table 1. (**C**) A protein–protein association network analysis conducted using the STRING database, illustrating the connections between known DC/TBD genes and the fusion genes listed in Table 1.

In a recent study, Lin and colleagues introduced QTIP-iPOND (Quantitative Telomeric Chromatin Isolation Protocol followed by Isolation of Proteins On Nascent DNA), a novel methodology for analyzing the replicating telomere proteome. They employed TRF1/TRF2 immunoprecipitation to isolate the telomere proteome and implemented EdU treatment, which labels nascent DNA at replication forks to specifically target the replicating telomere proteome. This approach led to the identification of multiple proteins significantly enriched in both telomeres and replicating telomeres [46]. Considering that DC is a Telomere Biology Disorder (TBD), we sought to determine if there was any correlation between our RNA-level gene fusion findings and this high-throughput proteomics data. We found that the protein products of certain genes, which were identified in DC-specific gene fusions (Table 1), show notable enrichment in telomeres. These genes include *ARHGAP17*, *CUL1*, *METAP1*, *PPA2*, *PSMB1*, *QKI*, *RRM2B*, *YLPM1*, and *ZNF638*, all of which have a significant presence in the telomere region (Figure 2A). Intriguingly, a subset of these genes, specifically *CUL1*, *METAP1*, *PPA2*, *PSMB1*, and *YLPM1*, are also prevalent in replicating telomeres (Figure 2B). Further analysis using the STRING database to explore known interactome data has expanded our understanding significantly. This analysis revealed that multiple genes implicated in DC-specific gene fusions are also associated with genes known to cause DC/TBD (Figure 2C).

### 2.3. Chimeric Protein Proteotoxicity as a Potential Driver of BMF in DC

As a consequence of these gene fusions, there is a potential emergence of unstable, disordered, and unfolded chimeric proteins. Such aberrant proteins can introduce proteotoxic stress within cells, potentially disrupting normal cellular functions and leading to pathological conditions [47]. Cellular proteostasis is essential for normal development, environmental stress resistance, infection management, and promoting healthy aging. Recent studies suggest that diverse proteostasis mechanisms play a crucial role in hematopoiesis, especially in erythropoiesis, and may function in a cell-type specific manner, particularly within hematopoietic stem cells (HSCs) [48,49]. The proteotoxic consequences of these disordered chimeric proteins highlight the significance of gene fusions, suggesting a potential molecular mechanism underlying the BMF observed in DC. To address this, CIDER was used to compute charge patterning and the propensity for disorder, aiming to predict the conformational and self-association behavior of the chimeric proteins resulting from the gene fusions listed in Table 1. These predictions were complemented using the FuzDrop and deePhase tools, which assess the propensity for phase separation, a characteristic feature of polyampholyte proteins—polymeric macromolecules with mixed cationic/anionic groups. The results emphasized the chimeric protein LRRFIP1-PTMA exhibited strong disordered and polyampholytic properties. Notably, a high propensity for phase separation is indicated by the FuzDrop and deePhase predictions (Table 2).

The N-terminal segment (1–32 aa) of the fusion gene is derived from the *LRRFIP1* gene, encoding the namesake protein, LRRFIP1. This protein plays a crucial role in the inflammatory stress response and mediates platelet activity [24,25,52]. Conversely, the *PTMA* gene, which encodes Prothymosin-A and comprises a significant portion of the fusion gene (33–128 aa), is also implicated in the inflammatory stress response. Further functions of Prothymosin-A include facilitation of the nuclear transport of proteins, exhibition of anti-apoptotic functions, and association with various cancers due to its cell growth-promoting activities [53].

**Figure 3:**
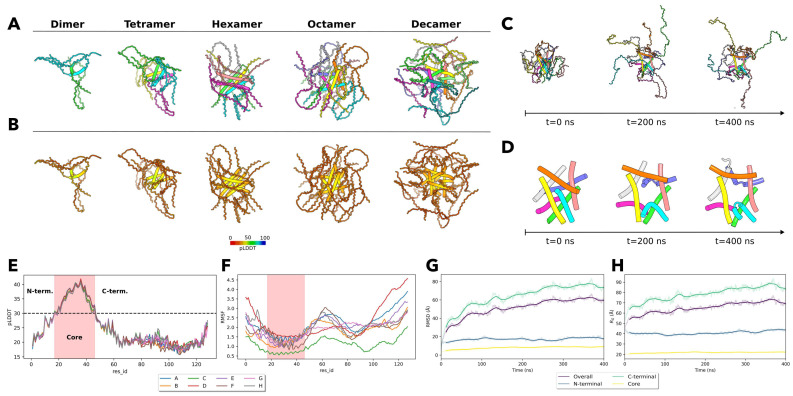
Self-association of LRRFIP1-PTMA fusion protein. (**A**) The topmost row categorizes the complexes from dimer to decamer with each chain uniquely colored, elucidating the assembly of the complexes. (**B**) In the subsequent row, the identical structures are portrayed, but they are colored based on their pLDDT scores, highlighting the well-structured helical core amidst the predominantly disordered protein. (**C**) The visualization presents three frames from the MD simulation of the octamer complex. The alpha-helical structured core is centrally located, with the notably flexible disordered regions dynamically adjusting around it. The individual chains of the complex determine the color scheme. (**D**) To further emphasize the minimal deviations of the ordered core from its initial state, compared to the more variable disordered regions, the trajectory exclusively showcases the ordered core. (**E**) The subfigure displays the pLDDT score for each residue of the predicted structure, plotted against the residue IDs. Instead of representing the entire structure, individual curves for each chain within the octamer are illustrated. Utilizing a pLDDT threshold of 30, the alpha-helical core structure, as depicted in Figure 1B, is demarcated. (**F**) The plot presents the Root Mean Square Fluctuation (RMSF) from the MD simulation, separated by chains. The analysis reveals that the structured region of the complex exhibits the least fluctuation. (**G**) The subfigure presents the Root Mean Square Deviation (RMSD) of the trajectory plotted against time. The trajectory is segmented into the overall structure (purple line), regions to the N-terminal (blue line) and C-terminal (green line) of the core, and the structured core (yellow line) itself. The RMSD for each segment is delineated, emphasizing a notably stable and less varied RMSD value for the core region in contrast to other parts of the structure. (**H**) The plot illustrates the radius of gyration (Rg) for these defined segments. Notably, the curve representing the core region remains the most consistent with minimal variation. In both Figure 1G,H, it is evident that the structural dynamics of the C-terminal region dominate the time evolution, closely mirroring the trends observed when evaluating RMSD and radius of gyration for the entire structure.

Given the absence of resolved structures for the unique LRRFIP1-PTMA fusion protein, its in silico self-association behavior must be evaluated using computationally generated higher-order oligomer structures. To achieve this, we employed AlphaFold 2 (AF2) multimer modeling, currently heralded as one of the most advanced methods for complex prediction. Its accuracy significantly surpasses traditional docking techniques, lending credibility to the resulting structures as better models for the protein’s natural complex formation [54]. However, historically limited accuracy of docking algorithms implies that newer and superior methods might offer only relative improvements. Nevertheless, the complexes depicted in Figure 3A,B align with anticipated characteristics of the fusion protein. The computational folding of incrementally added LRRFIP1-PTMA units yields a structure consistent with an intrinsically disordered protein complex. This complex features an alpha-helical core structure, with the remainder of the protein remaining unstructured. While the overall prediction scores are not exceptionally high, the score distribution remains consistent across all complexes. A medium-low pLDDT score, ranging between 30 and 40, characterizes the structured core, with the flexible regions scoring lower. These computational findings hint at the potential for further addition of LRRFIP1-PTMA units. However, it is noted that the accuracy of complexes generated by AF2 tends to decrease with an increasing number of subunits, even for homo-oligomers [54]. Consequently, beyond a certain subunit threshold, it becomes counterproductive to continue complex prediction. This study halted predictions upon reaching a decamer. Intriguingly, the overall score did not diminish as markedly as anticipated.

Given the moderate scores of the generated complexes and the general declining accuracy of AF2 modeling, a Molecular Dynamics (MD) simulation was executed to explore the dynamics of the created octamer structure. By incorporating this protein complex into a simulation box filled with explicit water and conducting an all-atom MD simulation, a refined physics-based simulation tool is employed. In this setting, a wholly unrealistic complex structure would likely not remain stable, thereby invalidating the constructed model. While a stable outcome in an MD simulation does not affirm the model’s absolute accuracy, it does serve as a preliminary investigation pathway. This strategy circumvents the need for computationally demanding free-energy simulations and has been previously effective in distinguishing native structures from decoy structures [55].

Figure 3C,D showcase the results of the conducted MD simulation. Three frames, representing the start, midpoint, and end of the simulation, are depicted in Figure 1C to portray the dynamic behavior. Here, the alpha-helical core remains intact, undergoing minor reorganization, as depicted in Figure 3D, but largely retaining its initial form. In contrast, other protein segments exhibit significant flexibility, especially within the disordered charged regions, aligning well with anticipated behaviors for these sections.

While the simulation cannot conclusively demonstrate aggregation or phase separation behaviors for this protein, it suggests the modeled higher-order oligomer structure is stable. The observed dynamic behavior—featuring a stable core and flexible arms—aligns seamlessly with the inherently disordered nature of this complex.

Figure 3E quantitatively presents the pLDDT score for each chain against its residue ID. The protein is segmented into N-terminal, core, and C-terminal sections, determined by a pLDDT threshold of around 30. The MD simulation-derived per-residue fluctuation is similarly visualized in Figure 3F, depicting the RMSF for each chain. Here, RMSF values are lowest within the core region and peak in the C-terminal side of the structure, housing the elongated disordered regions.

Standard metrics utilized for assessing protein stability within MD simulations, namely, RMSD and the radius of gyration Rg, are highlighted in Figure 1G and 1H, respectively. The different curves visible for both metrics are due to calculating the respective metrics for the entire complex versus only for the N-terminal, Core or C-terminal part of the complex. Evidently, the core region exhibits the highest stability, with overall fluctuations dominated by movements in the C-terminal region.

These analyses indicate that within the simulation, the core region, encompassing the fusion breakpoint site, adopts an alpha-helical conformation conducive to self-association. This observation emphasizes the complex’s intrinsic stability and dynamic behavior, aligning with the anticipated properties derived from the fusion protein’s pronounced polyampholyte characteristics. This might propel a unique fusion breakpoint-induced self-association, which could pose challenges to the cellular proteostasis mechanisms through potential aggregation, amyloidogenic fibril formation, or aberrant phase separation. Such disruptions are known to instigate hematopoietic abnormalities and an array of rare genetic disorders [56,57,58].

## 3. Materials and Methods

### 3.1. Dyskeratosis Congenita RNA-Seq Data

Pulmonary fibrosis is a well-documented manifestation of Dyskeratosis Congenita (DC), presenting significant clinical challenges [59,60,61,62]. To comprehensively address the genomic and pathological burdens associated with DC, our study focused on analyzing data obtained from lung epithelial cells. The dataset comprised 143 single-cell RNA-Seq samples obtained from lung epithelial cells, including 48 samples from patients with DC and 95 samples from healthy individuals [63]. RNA-Seq raw data were sourced from the Gene Expression Omnibus (GEO) at the National Center for Biotechnology Information (NCBI). To retrieve the associated Sequence Read Archive (SRA) files, the GEO accession number GSE83501 was referenced. Subsequently, these SRA files were converted to the fastq format using the fasterq-dump command from the SRA Toolkit. Prior to any downstream processing, the quality of the fastq files was assessed with FastQC to ensure data robustness.

### 3.2. Gene Fusion Analysis

For the identification and assessment of gene fusions in the DC RNA-Seq dataset, Arriba was employed, a streamlined and accurate RNA-seq aligner and fusion detector [8]. Initially, FastQC was used to assess the quality of raw sequencing reads. Following this, Trimmomatic was utilized to trim the reads for both quality and adapter sequences. These quality-controlled reads were mapped to the human reference genome (GRCh38) using the STAR aligner. Arriba was executed alongside STAR, taking advantage of STAR’s capability to process spliced alignments for enhanced fusion identification. The output from Arriba was curated to retain only high-confidence fusions, discarding artifacts and fusions with insufficient read support through its integrated filtering script. These retained fusions were annotated using the Ensembl database, providing details about the implicated genes and anticipated protein derivatives. Finally, high-confidence fusions were scrutinized by considering non-zero split read and discordant mate counts to minimize potential false-positive results. Visualization of the gene fusions was performed using the shinyCircosV2 tool [64].

### 3.3. Gene Ontology Analysis

To elucidate the functional consequences of the gene fusions, a Gene Ontology (GO) analysis utilizing the PANTHER (Protein ANalysis THrough Evolutionary Relationships) classification system, an online bioinformatics resource, was conducted [65]. This involved uploading the compilation of unique genes implicated in the fusions, as detected by the Arriba tool, to the PANTHER server. The analysis focused on the Biological Process category of the GO terms, which were then systematically annotated to our dataset. The primary objective was to assess the enrichment of these GO terms in our gene set relative to a baseline reference, which encompassed the entire human gene repertoire. This comparative analysis was statistically validated using the False Discovery Rate correction applied to Fisher’s exact test results, thereby refining the p-values for accuracy. GO terms that exhibited an adjusted p-value below the threshold of 0.05 were deemed to be significantly enriched, indicating a notable overrepresentation in our gene fusion dataset.

### 3.4. Overrepresentation and Network Analyses

An overrepresentation analysis (ORA) of the Web-based Gene Set Analysis Toolkit (WebGestalt) was employed to elucidate the potential functional enrichments of genes involved in gene fusions [66]. WebGestalt was configured to conduct the ORA against the predefined gene sets of TCGA RNA-Seq hierarchical co-expression modules, specifically focusing on the hallmark gene sets which encompass a defined set of studied cancer types. The parameters were set to employ the hypergeometric test for statistical significance and the Benjamini–Hochberg procedure was utilized for the correction of multiple testing with a false discovery rate (FDR) threshold of 0.05. The networks of overrepresented gene sets were plotted using the integrated network visualization tool within WebGestalt.

### 3.5. Protein–Protein Association Network Analysis

The protein–protein association network was analyzed using the STRING database v12.0, a comprehensive resource compiling known and predicted protein interactions [67]. Our gene list, including the known DC/TBD (Dyskeratosis Congenita and related Telomere Biology Disorders) genes [68] together with identified DC-specific fusion genes (Table 1), was inputted with a confidence score threshold set at medium (0.4) to ensure interaction significance. STRING generated an interaction network, where nodes represent proteins and edges denote interactions, with edge thickness indicating the strength of data support.

### 3.6. Disorder, Polyampholyte, and Self-Association Propensity Analysis

For the analysis of intrinsically disordered regions (IDRs) and their polyampholyte propensity in gene fusions associated with DC, the CIDER (Classification of Intrinsically Disordered Ensemble Regions) bioinformatics tool was employed. This tool provided an in-depth assessment based on charge patterning within the IDRs. Gene fusion sequences, curated from the DC-associated dataset, were input into the CIDER webserver v2.0 or localCIDER v0.1.20. The software generated the Das–Pappu diagram by computing kappa, the fraction of charged residues, the net charge per residue, hydropathy, and disorder-promoting descriptors to elucidate potential polyampholyte behavior [50,51].

The FuzDrop webserver was utilized to estimate the likelihood of liquid–liquid phase separation (LLPS), characteristic of polyampholyte proteins [69,70,71]. This prediction is grounded in a machine learning algorithm trained with experimental datasets from sequence features of proteins documented to exhibit LLPS. High scores correspond to an increased predisposition toward LLPS behavior [70]. To strengthen the LLPS prediction, we also used the deePhase tool. This tool leverages machine learning models through neural network-based sequence analysis, taking into account both biophysical and sequence-specific characteristics of phase-separating proteins [72].

### 3.7. Prediction of Fusion Protein Tertiary Structure

For the fusion protein Lrrfip1-Ptma, homo-oligomer structures were generated using multimer modeling in AlphaFold version 2.3 (AF2) [73]. Complexes were systematically assembled in increments, ranging from dimers to decamers. The date cutoff parameter was set to 24 May 2022, ensuring that structures available in the PDB up to this date were utilized as input templates. From each AF2 multimer run, 25 models were generated, of which only the top-ranked model was selected. These chosen structures were subsequently aligned with each other for visualization purposes, employing the PyMol software v2.5.0. [74]

### 3.8. Molecular Dynamics Simulations and Analysis

A Molecular Dynamics (MD) simulation spanning 400 ns was conducted on the LRRFIP1-PTMA fusion protein octamer generated by AF2 using the GROMACS platform [75,76,77,78,79,80,81,82], employing the CHARMM36 force field [83]. The protein was positioned within a dodecahedron, subject to periodic boundary conditions, and ions were introduced to neutralize its overall charge. The MD simulation protocol followed steps 0 to 5 as delineated at https://github.com/carlocamilloni/labtools/tree/main/mdps/atomistic (accessed between September and October 2023).

For further clarity, the initial protein configuration underwent three sequential minimization steps. The process began with the steepest descent method, a straightforward minimization technique. This was succeeded by the conjugate gradient and LBFSG minimization methods [84,85], which are comparatively more sophisticated. Beginning with a basic minimizer and feeding its output into the subsequent advanced minimizers offers a heuristic approach to achieving a more optimized state for the preparatory equilibration steps preceding the actual simulation. A set of relaxation and preparatory phases commences from the minimized configuration, culminating in the production run utilizing a modern method that facilitates more direct simulation in the NPT ensemble [86]. The NPT ensemble defines the thermodynamic conditions, dictating that the number of particles (N), pressure (P), and temperature (T) remain constant. Notably, the NPT simulation operated at a temperature of 300 K and a pressure of 1 bar, standard conditions for MD simulations, and also the conditions under which force field parameterization occurs.

To analyze the resulting MD simulation, a specialized Python script, underpinned by the biotite library [87,88], was employed. This script facilitated the calculation of metrics such as the Root Mean Square Deviation (RMSD), radius of gyration (Rg), and the Root Mean Square Fluctuation (RMSF) based on the simulation trajectory. Additionally, the pLDDT score of the input structure was graphically represented, highlighting the structured core contingent on a pLDDT score threshold. This analytical tool is also extensively dependent on the Scipy [89], NumPy [90], and matplotlib [91] libraries.

## 4. Discussion

A hallmark of DC at the cellular level is telomere dysfunction and subsequent telomere shortening. Telomeres, the protective end caps of chromosomes, play a crucial role in maintaining chromosomal integrity and stability. When these telomeres are shortened, as seen in DC, they lose their protective capability, leading to the fusion of chromosomal ends, genomic instability, and heightened risk of cell cycle arrest or cell death. This genomic instability due to telomere shortening in DC is believed to be a significant contributing factor to the increased susceptibility to cancers, bone marrow failure, and other degenerative disorders often observed in affected individuals [92].

Genomic instability, marked by an enhanced susceptibility to genetic modifications, is instrumental in a vast array of diseases. Within the hematopoietic system, the bone marrow stands as the chief location for blood cell generation, creating intricate connections between genomic instability and BMF. Fundamentally, hematopoietic stem cells (HSCs) situated in the bone marrow birth all blood cell categories. Any disruptions in their genomic structure can critically hinder their growth and differentiation potentials. A notable feature of DC is the dysfunction and reduction in telomeres, which act as primary contributors to genomic instability in the marrow. As time progresses, this instability can give rise to chromosomal irregularities, genetic reshufflings, DNA mutations, and defective DNA repair mechanisms in hematopoietic cells. In acute instances, these anomalies can instigate clonal proliferation that poses the threat of progressing into blood disorders like MDS or AML. In our research, we pinpointed several genes that result in gene fusions (Table 1). These genes, in their original states, predominantly serve to uphold genomic stability and ensure the equilibrium of platelet and lymphocyte activities. Their dysregulation or inhibition can wreak havoc, influencing a plethora of crucial pathways and mechanisms. These include DNA damage triggered by hypertranscription or via ATM-P53 axis, arrest in G1 and S-phase of the cell cycle, HR and NHEJ defects due to the suppression of BRCA1-RAD51 and HDAC1 activation, instigating T-cell acute lymphoblastic leukemia by modifying the NOTCH1, PTEN-PI3K-AKT, and MYC pathways, curtailing platelet function by impacting cyclic nucleotide pathways, undermining hemostasis by inhibiting platelet clumping through the reduction in αIIbβ3 expression, obstructing DNA replication due to changes in the RBBP6/ZBTB38/MCM10 axis, and imbalancing the proliferation-to-apoptosis ratio of lymphocytes, among others [9,12,13,14,15,16,17,18,19,20,21,22,23,24,25,26,27,28,29].

Tumor suppressor genes, pivotal in controlling cell growth and preventing malignant transformations, often experience alterations leading to cancer development. One intriguing phenomenon is their involvement in gene fusions that can disrupt the normal functioning of tumor suppressors and propel cancer progression. This interplay between tumor suppressor genes and gene fusions forms a crucial nexus in malignant transformation, offering novel avenues for research and therapeutic interventions. Within this study, the identified functions of these tumor suppressor genes span a range of cellular processes: they regulate the WNT/β-CATENIN pathway, activate the STING pathway, inhibit epithelial-to-mesenchymal transition, enhance the efficacy of immunotherapy via the PYK2/TAZ/PDL1 pathway, oversee ribosomal RNA maturation, and control tumor growth through the RBMS3/TWIST1/MMP2 pathway [31,32,33,34,35,36,37,38,39,40,41,42,43,44]. These multifaceted roles emphasize the clinical significance of comprehending the molecular mechanisms of these genes. Notably, in DC patients, the dysregulation of these genes due to gene fusion events could potentially drive the onset of BMF-associated malignancies.

Genomic mapping of the rearrangements of both healthy and DC groups revealed a highly heterogeneous distribution of gene fusion events with no significant enrichment at telomeric regions in DC samples (Figure 1A). This observation is initially surprising. Given the known role of telomere dysfunction in driving genomic instability, one might expect an increased frequency of genomic rearrangements, particularly at telomeric regions, as a hallmark of the disease. However, the nature of DC and its developmental context offer a plausible explanation for these findings. DC, being a congenital genetic disorder, implies that the genomic instabilities associated with telomere dysfunction could manifest differently compared to acquired telomere diseases. In early developmental stages, such as in the embryo, telomere dysfunction might indeed drive enriched genomic rearrangements at telomeric regions. However, in later stages of DC (as in this study), the disease’s congenital aspect could mean that these rearrangements occur in a more distributed manner across the genome. The escalation of genomic instability over the course of development not only shortens telomeres; it also involves the alteration of genes responsible for maintaining genomic stability (Table 1). This includes genes involved in DNA repair, replication, and chromatin remodeling. The malfunctioning of these critical pathways could lead to a more generalized form of genomic instability, rather than one localized to telomeric regions.

We have also found that the protein products of genes involved in DC-specific gene fusions are significantly enriched in telomeres, and more specifically, some in replicating telomeres (Figure 2A,B). This evidence implies that gene fusion events could potentially disrupt these genes’ inherent role in telomere protection and replication. Further interactome analysis has shown that a number of genes implicated in DC-specific gene fusions are linked with genes known to be associated with DC/TBD (Figure 2C). This correlation not only reinforces our hypothesis but also highlights the critical nature of these genes in maintaining telomere integrity and in regulating essential DC/TBD-causing genes through direct interactions.

Given that the *LRRFIP1* and *PTMA* genes are situated at the distal end of the q arm of chromosome 2, and considering that telomere dysfunction and shortening are defining characteristics of DC, it is conceivable that these chromosomal regions in DC patients are highly susceptible to rearrangements as a consequence of telomere dysfunction-driven genomic instability. Moreover, the *LRRFIP1* and *PTMA* genes have previously been identified in fusion events with various genes, playing roles in the pathogenesis of several malignancies. Specifically, in leukemias, *PTMA* has been reported in fusion with *OAZ1* and *CXCR4*. *LRRFIP1* has been implicated in 8p11 myeloproliferative syndrome through its fusion with *FGFR1*, in inflammatory myofibroblastic tumors with *ALK* and in atypical Spitz tumors with *MET* [27,93,94,95,96].

Further computational analyses shed light on the structural and biophysical attributes of the LRRFIP1-PTMA fusion protein, revealing it to be a predominantly disordered yet potent polyampholyte (Table 2). Deep learning-based protein structure prediction and molecular dynamic simulations substantiated its propensity to form higher-order oligomers via self-association (Figure 3). Given prior research indicating Prothymosin-A’s inherent disorderliness and inclination to form amyloidogenic fibrils in acidic environments [97,98], it is reasonable to deduce that the LRRFIP1-PTMA fusion, composed predominantly by Prothymosin-A, could elicit proteotoxic effects. This could manifest either as the formation of amyloidogenic fibrils or aberrant phase-separated biomolecules, contingent upon the cellular context. Such exacerbated proteotoxic challenges, by perturbing proteostasis, might jeopardize the vitality and efficacy of hematopoietic cells, potentially culminating in BMF and related malignancies.

**Figure 4:**
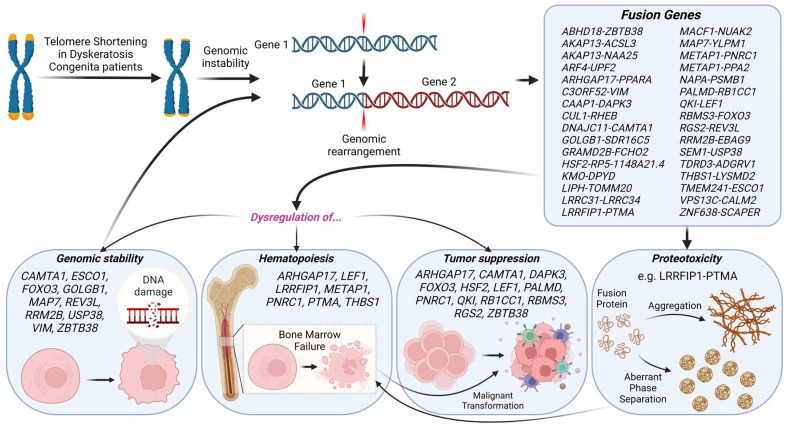
A comprehensive summary of deciphering the path from telomere dysfunction to hematopoietic challenges in DC. The figure depicts the cascade from telomere shortening and genomic instability to gene rearrangements, emphasizing the dysregulation of crucial genes through the emergence of fusion genes, and their potential inhibitory and proteotoxic implications resulting in detrimental consequences such as BMF and malignant transformation.

In summary, our work suggests that the central feature of DC, telomere dysfunction and shortening, can lead to a severe risk of genomic instability, which in turn can disrupt the regulation of key genes responsible for genomic stability, hematopoiesis, and tumor suppression. Such disturbances can pave the way for BMF, genomic alterations, and increased genomic instability, further elevating the risk of malignant transformations due to compromised tumor suppressor genes. Additionally, gene fusions arising from genomic instability-induced rearrangements can result in the creation of distinct fusion proteins. These proteins, with their potential for proteotoxic stress via self-association, can further contribute to BMF risks (Figure 4). Developing small molecules or biologics that precisely target the breakpoints of fusion genes, or the self-association regions of the ensuing fusion proteins, could attenuate their detrimental impact. When integrated with bone marrow transplantation or androgen therapy, such strategies may offer a synergistic approach to decelerate disease progression.

However, recognizing the importance of discussing the limitations of our methodologies and analyses is crucial for providing a balanced and comprehensive understanding of our study. In our analysis, we utilized the Arriba tool for gene fusion detection in RNA-Seq data [8]. Arriba has demonstrated high efficiency in detecting true positive gene fusions, as evidenced by its favorable performance in a recent comprehensive gene fusion detection benchmark analysis [99]. However, it is important to acknowledge that no analytical tool is without limitations. A notable limitation of Arriba, shared by many gene fusion detection tools, is the disparity in coverage calculations, which include duplicates, as opposed to supporting reads that do not. Consequently, the coverage values and supporting read counts are only approximately comparable, particularly in scenarios where a high number of duplicates is anticipated, such as with targeted sequencing libraries or in the case of highly expressed genes [8]. To mitigate this and enhance the true detection efficiency in our analysis, we applied a lower threshold value (≥1) for split reads, discordant mates, and coverage in the evaluation of Arriba’s gene fusion output. Additionally, Arriba shows a deficiency in accurately detecting deletions, which are often indistinguishable from normal splicing in RNA-Seq data. This challenge could potentially be addressed by integrating RNA-Seq data with whole-genome sequencing or by employing additional algorithms that focus on differential exon expression and indel detection.

While acknowledging the significance and utility of computational analyses in predicting high-confidence gene fusion candidates, their associated pathways, and resultant fusion proteins, along with their potential roles in disease progression—thereby streamlining the identification of key candidates and reducing potential experimental workload—it is imperative to experimentally verify these detected gene fusion events and their protein products. Such experimental validation is essential not only for confirming the accuracy of computational predictions but also for gaining insights into the functional relevance of these fusions, establishing their exact links with the DC disease mechanisms, and identifying possible therapeutic targets.

## Figures and Tables

**Table 1:** A comprehensive summary of DC-specific in-frame gene fusions.

Gene 1	Gene 2	Breakpoint 1	Breakpoint 2	Type	Split Reads 1	Split Reads 2	Discordant Mates
*ABHD18*	*ZBTB38*	4:128011700	3:141445718	translocation	15	22	19
*AKAP13*	*ACSL3*	15:85669830	2:222922708	translocation	4	2	1
*AKAP13*	*NAA25*	15:85645954	12:112039339	translocation	12	22	11
*ARF4*	*UPF2*	3:57575548	10:12035441	translocation	3	2	7
*ARHGAP17*	*PPARA*	16:24964197	22:46218263	translocation	2	9	4
*C3ORF52*	*VIM*	3:112093489	10:17236294	translocation	8	8	7
*CAAP1*	*DAPK3*	9:26884810	19:3969829	translocation	5	20	15
*CUL1*	*RHEB*	7:148767749	7:151467211	inversion	2	4	7
*DNAJC11*	*CAMTA1*	1:6701729	1:6820181	inversion	4	5	2
*GOLGB1*	*SDR16C5*	3:121722262	8:56309027	translocation	12	14	11
*GRAMD2B*	*FCHO2*	5:126371567	5:72989427	duplication	8	8	14
*HSF2*	*RP5-1148A21.4*	6:122423686	6:63576436	duplication	1	3	13
*KMO*	*DPYD*	1:241532498	1:97193248	inversion	1	4	4
*LIPH*	*TOMM20*	3:185552423	1:235122372	translocation	3	6	6
*LRRC31*	*LRRC34*	3:169854813	3:169808745	deletion/read-through	17	11	16
*LRRFIP1*	*PTMA*	2:237627740	2:231711348	duplication	2	6	5
*MACF1*	*NUAK2*	1:39388658	1:205311825	inversion	14	11	31
*MAP7*	*YLPM1*	6:136550342	14:74829213	translocation	5	9	5
*METAP1*	*PNRC1*	4:98995867	6:89083753	translocation	6	8	11
*METAP1*	*PPA2*	4:99043387	4:105399164	inversion	6	1	5
*NAPA*	*PSMB1*	19:47514843	6:170549113	translocation	1	2	4
*PALMD*	*RB1CC1*	1:99646362	8:52658976	translocation	2	1	3
*QKI*	*LEF1*	6:163535125	4:108089257	translocation	8	14	15
*RBMS3*	*FOXO3*	3:29281756	6:108663455	translocation	7	11	6
*RGS2*	*REV3L*	1:192809181	6:111405630	translocation	5	1	2
*RRM2B*	*EBAG9*	8:102212776	8:109550810	inversion	2	1	1
*SEM1*	*USP38*	7:96694798	4:143195716	translocation	2	2	1
*TDRD3*	*ADGRV1*	13:60467379	5:90756979	translocation	7	9	19
*THBS1*	*LYSMD2*	15:39587520	15:51725121	inversion	20	17	29
*TMEM241*	*ESCO1*	18:23437781	18:21540700	deletion	3	5	3
*VPS13C*	*CALM2*	15:62044212	2:47170764	translocation	1	4	6
*ZNF638*	*SCAPER*	2:71350271	15:76665789	translocation	2	2	2

Column descriptions: Gene1 and Gene2: ‘Gene1’ represents the gene contributing the 5′ end of the transcript, while ‘Gene2’ represents the gene contributing the 3’ end. Breakpoint1 and Breakpoint2: These columns display the genomic coordinates where the breakpoints occur in ‘Gene1’ and ‘Gene2’, respectively. Type: This column describes the kind of genomic event, inferred from the orientation of supporting reads and breakpoint coordinates. Possible events include Translocation (occurring between different chromosomes), Duplication, Inversion, Deletion, and Read-Through (deletions within intron size (<400 kb)). Split_reads1 and Split_reads2: These represent the count of split fragments anchored in ‘Gene1’ or ‘Gene2’. The gene aligned with the larger segment of the split read is termed the anchor. Discordant_mates: This column tallies the number of paired fragments, also known as spanning or bridge reads, supporting the fusion event. For detailed explanations refer to Uhrig et al. (2021) [8].

**Table 2:** Predictions of charge patterning, intrinsic disorder, and phase separation propensity for in-frame fusion proteins.

Gene Fusion	Length (aa)	κ	FCR (≥0.35)	NCPR	Hydropathy	Disorder Promoting	Plot Region(≥3)	FuzDrop P_LLPS_	deePhase(≥0.5)
*ABHD18-ZBTB38*	242	0.162	0.273	0.033	3.867	0.599	2	0.2472	0.37
*AKAP13-ACSL3*	1714	0.162	0.239	−0.077	3.973	0.714	1	1.0000	0.83
*AKAP13-NAA25*	1584	0.163	0.238	−0.07	4.032	0.704	1	1.0000	0.84
*ARHGAP17-PPARA*	536	0.208	0.276	0.011	4.146	0.597	2	0.2019	0.31
*C3ORF52-VIM*	131	0.196	0.244	−0.092	4.292	0.603	1	0.9178	0.26
*CUL1-RHEB*	391	0.141	0.274	−0.008	3.925	0.614	2	0.2704	0.24
*DNAJC11-CAMTA1*	1682	0.197	0.205	−0.006	4.014	0.672	1	0.9994	0.86
*GOLGB1-SDR16C5*	370	0.192	0.259	−0.043	4.144	0.643	2	0.6604	0.53
*HSF2-RP5-1148A21.4*	423	0.223	0.267	−0.054	3.884	0.619	2	0.5350	0.67
*KMO-DPYD*	229	0.239	0.227	0.009	4.381	0.581	1	0.1621	0.16
*LIPH-TOMM20*	120	0.261	0.242	−0.008	4.413	0.617	1	0.1939	0.19
*LRRC31-LRRC34*	747	0.203	0.216	−0.017	4.326	0.564	1	0.1573	0.57
*LRRFIP1-PTMA*	128	0.417	0.562	−0.328	2.505	0.852	3	0.9949	0.66
*MACF1-NUAK2*	5828	0.148	0.276	−0.036	3.945	0.654	2	0.9924	0.81
*MAP7-YLPM1*	114	0.164	0.412	−0.009	3.366	0.772	3	0.3332	0.35
*METAP1-PNRC1*	185	0.152	0.238	0.065	3.76	0.649	1	0.5152	0.52
*METAP1-PPA2*	334	0.195	0.254	−0.003	3.996	0.626	2	0.2324	0.38
*NAPA-PSMB1*	236	0.128	0.246	0	4.363	0.619	1	0.2091	0.089
*PALMD-RB1CC1*	1046	0.117	0.324	−0.056	3.81	0.649	2	0.7123	0.81
*QKI-LEF1*	443	0.16	0.26	0.029	3.718	0.677	2	0.9907	0.73
*RBMS3-FOXO3*	491	0.277	0.167	−0.012	3.879	0.676	1	0.9996	0.85
*RGS2-REV3L*	3032	0.197	0.254	0.012	3.847	0.647	2	0.9999	0.84
*SEM1-USP38*	826	0.213	0.215	−0.044	4.185	0.61	1	0.6913	0.8
*TDRD3-ADGRV1*	2459	0.199	0.193	−0.053	4.562	0.593	1	0.6100	0.57
*TMEM241-ESCO1*	74	0.31	0.203	0.014	4.526	0.5	1	0.1198	0.17
*VPS13C-CALM2*	196	0.179	0.316	−0.133	4.032	0.612	2	0.1353	0.1
*ZNF638-SCAPER*	1003	0.198	0.2	0.011	4.086	0.616	1	0.8597	0.77

For this analysis, fusion hits with low-quality breakpoint peptide sequences were excluded to enhance the accuracy and true positivity of the results. For a comprehensive description of the parameters and plot regions, please consult Das and Pappu (2013) and Holehouse et al. (2017) [50,51].

## Data Availability

All data generated or analyzed during this study are included in this published article (and its supplementary information files).

## Supplementary Materials

The following supporting information can be downloaded at: https://www.mdpi.com/article/10.3390/ijms25031606/s1.
