# Supplementary figures and images for: Linking Gene Fusions to Bone Marrow Failure and Malignant Transformation in Dyskeratosis Congenita
Source: Int J Mol Sci. 2024 Jan 28;25(3):1606. doi: 10.3390/ijms25031606 (PMC10855549; doi:10.3390/ijms25031606)

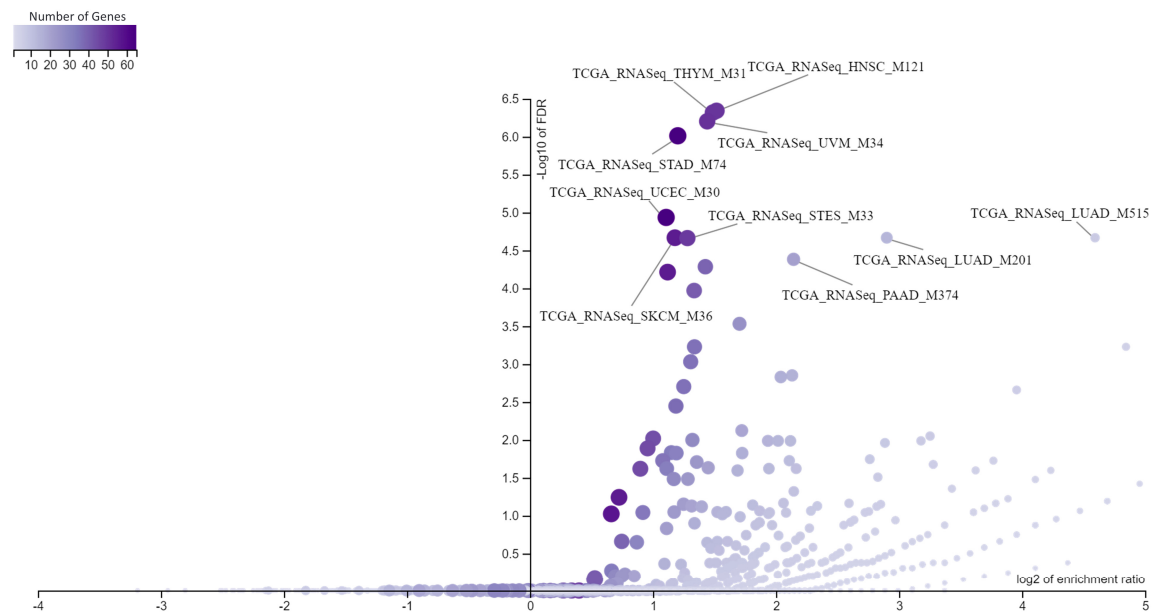

**Figure S1.** Fusion genes significantly enriched in many cancers

Supplement: Supplementary file 1 [file ijms-25-01606-s001.zip › Supplementary_Figure1_Revision.pdf]
